# Supplementary material for: Early Application of ECMO after Sudden Cardiac Arrest to Prevent Further Deterioration: A Review and Case Report
Source: J Clin Med. 2023 Jun 25;12(13):4249. doi: 10.3390/jcm12134249 (PMC10342643; doi:10.3390/jcm12134249)
Supplement: Supplementary file 1 [file jcm-12-04249-s001.zip › jcm-2384365-supplementary.pdf]

SAVE (Survival after Veno-Arterial ECMO) Score

RESULT SUMMARY:
7 points
SAVE Score

Class I
Risk class

75 %
In-hospital survival

INPUTS:
Age, years —> 7 = 18-38
Weight —> 2 = 143-196 lbs (65-89 kg)
Myocarditis —> 0 = No
Refractory VT/VF —> 0 = No
Post heart or lung transplantation —> 0 = No
Congenital heart disease —> 0 = No
Acute renal failure —> 0 = No
Chronic renal failure —> 0 = No
HCO_3 before ECMO ⩽15 mmol/L (91.5 mg/dL) —> 0 = No
Duration of intubation prior to initiation of ECMO, hrs —> 0 = ⩽10
Peak inspiratory pressure ⩽20 cm H_2O (⩽2.0 kPa) —> 3 = Yes
Pre-ECMO cardiac arrest —> -2 = Yes
Diastolic blood pressure before ECMO ⩾40 mmHg —> 3 = Yes
Pulse pressure before ECMO ⩽20 mmHg —> 0 = No
Liver failure —> 0 = No
CNS dysfunction —> 0 = No
